# Monocyte Dynamics in Chikungunya Fever: Sustained Activation and Vascular-Coagulation Pathway Involvement

**DOI:** 10.3390/v17091224

**Published:** 2025-09-07

**Authors:** Caroline Fernandes dos Santos, Priscila Conrado Guerra Nunes, Victor Edgar Fiestas-Solorzano, Mariana Gandini, Flavia Barreto dos Santos, Roberta Olmo Pinheiro, Luís Jose de Souza, Paulo Vieira Damasco, Luzia Maria de Oliveira Pinto, Elzinandes Leal de Azeredo

**Affiliations:** 1 Laboratório das Interações Vírus Hospedeiros, Instituto Oswaldo Cruz, Rio de Janeiro 21040-360, Brazil; carol.uned@gmail.com (C.F.d.S.); priscila.nunes87@gmail.com (P.C.G.N.); vicfiso@gmail.com (V.E.F.-S.); flaviab@ioc.fiocruz.br (F.B.d.S.); lpinto@ioc.fiocruz.br (L.M.d.O.P.); 2 Laboratório de Microbiologia Celular, Instituto Oswaldo Cruz, Rio de Janeiro 21040-360, Brazil; mariana.gandini@fiocruz.br; 3 Laboratório de Hanseníase, Instituto Oswaldo Cruz, Rio de Janeiro 21040-360, Brazil; robertaolmo@gmail.com; 4 Centro de Doenças Imuno-Infecciosas (CRDI), Faculdade de Medicina, Campos dos Goytacazes, Brasil Campos dos Goytacazes, Rio de Janeiro 28025-496, Brazil; luizjosedes@gmail.com; 5 Hospital Casa Rio Laranjeira, Rio de Janeiro 22240-000, Brazil; paulovieiradamasco@gmail.com; 6 Departamento de Medicina Geral, Universidade Federal do Estado do Rio de Janeiro (UERJ), Rio de Janeiro 20550-170, Brazil; 7 Departamento de Doenças Infecciosas, Universidade Federal do Estado do Rio de Janeiro (UniRio), Rio de Janeiro 21941-902, Brazil

**Keywords:** Chikungunya virus, monocyte subsets, TLR7 signaling, chronic inflammation, coagulation factors, vascular dysfunction

## Abstract

Chikungunya fever (CF), caused by the Chikungunya virus (CHIKV), is characterized by disabling symptoms such as joint pain that can last for months. Monocytes play a central role in immune modulation and viral replication during infection. This study evaluated the clinical and immunological profiles of patients with laboratory-confirmed CF. Fever and joint pain were the most frequently reported symptoms, whereas edema was more common in women. CHIKV-infect individuals exhibited increased TLR4 expression in non-classical monocytes (CD14+CD16++). Additionally, intermediate (CD14+CD16+) and non-classical (CD14+CD16++) monocytes expressing TLR7 were enriched during the acute phase and in some chronic patients, thereby suggest prolonged TLR7 pathway activation. Levels of soluble CD163 (sCD163)—a marker of monocyte/macrophage activation—were elevated as well, indicating sustained immune activation. Coagulation-related mediators—including Tissue factor (TF) and Tissue factor pathway inhibitor (TFPI)—also increased, despite the rarity of hemorrhagic events or thrombocytopenia. Patients with arthritis demonstrated higher frequencies of TLR7+ intermediate monocytes and elevated Epidermal growth factor (EGF) levels, whereas those with edema exhibit increased Vascular endothelial growth factor (VEGF) levels. Overall, these findings highlighted the differential activation of CD16+ monocytes and suggested that sCD163 is a marker of monocyte/macrophage activation during CHIKV infection.

## 1. Introduction

Chikungunya virus (CHIKV) is a mosquito-borne alphavirus that causes chikungunya fever (CF) and poses a significant global public health challenge. In particular, it is responsible for recurring epidemics in Brazil. The disease typically begins with an abrupt onset of fever lasting from several days to two weeks, accompanied by debilitating polyarthralgia. Pain and swelling—usually symmetrical—most commonly affect the hands, wrists, ankles, and feet, and can persist for months or even years [1]. Other common symptoms include maculopapular rashes covering the trunk and extremities, headache, fatigue, nausea, vomiting, depression, hair loss, conjunctivitis, and myalgia. Severe clinical complications have also been reported—including neurological, cardiac, and multiple organ failure [2].

Subcutaneous inoculation of CHIKV occurs through the bite of a mosquito from the *Aedes* genus, following which the virus spreads via the microvasculature and regional lymph nodes. It is introduced into the skin along with the saliva of the infected vector and subsequently infects various cells—such as fibroblasts, keratinocytes, and langerhans cells. Once disseminated throughout the body, the virus affects the bones, muscles, and joint tissues. Thereafter, it triggers the acute phase of the disease, which is strongly associated with a local inflammatory response [3,4]. The pathogenesis of CHIKV infection is influenced by several factors—including age, host immune and inflammatory responses, and virulence of the viral strain [5]. Furthermore, soluble factors secreted by macrophages amplify the inflammatory process by activating lymphocytes and natural killer (NK) cells [6]. The pathogenesis of infection is also associated with elevated levels of inflammatory mediators, such as cytokines and chemokines [2,7].

Monocytes/macrophages, plasmacytoid dendritic cells, and B lymphocytes have been identified as target cells for CHIKV infection [8,9,10,11]. In non-human primates, viral replication within the first 2–3 days post-infection is predominantly observed in the monocytes/macrophages infiltrating the draining lymph nodes [12]. In humans, persistence of CHIKV in synovial macrophages is associated with the development of chronic arthralgia [13]. Her et al. demonstrated the presence of the CHIKV antigen in monocytes from acutely infected patients and confirmed the susceptibility of circulating monocytes—isolated from peripheral blood mononuclear cells—to CHIKV infection in vitro [14]. More recently, human monocytes and monocyte-derived macrophages were shown to be permissive to infection and capable of actively contributing to antiviral responses by initiating innate immune activation [15].

Circulating human monocytes are classified into distinct subsets based on the ex-pression of the surface markers, CD14 and CD16. Classical monocytes (CD14++CD16−) represent approximately 90% of the total monocyte population and exhibit high CD14 expression, with no CD16 expression. The remaining 10% consist of intermediate monocytes (CD14+CD16+), which express both markers, and nonclassical monocytes (CD14+CD16++), which are characterized by lower CD14 and high CD16 expression [16]. Among these subsets, classical monocytes display strong phagocytic activity and are primarily involved in orchestrating inflammatory responses, thereby influencing the progression of various diseases. Intermediate monocytes are typically recruited during the later stages of inflammation and specialize in antigen presentation, whereas nonclassical monocytes play a key role in immune surveillance and antiviral responses [17]. In particular, CD16+ monocyte subsets—comprising both intermediate and nonclassical monocytes—are central players in the innate immune response. They act as potent producers of inflammatory cytokines, and express pattern recognition receptors (PRRs) such as Toll-like receptors (TLRs) 7 and 8, which are critical for viral recognition and immune activation [18]. Moreover, soluble mediators—such as growth factors (GFs), coagulation factors, and pro-inflammatory cytokines—may contribute to either the exacerbation of inflammation during the acute phase or its persistence in the chronic phase of CHIKV infection, both of which can ultimately manifest as joint pain [19].

Despite the recognized burden of joint pain and inflammation in CHIKV infection, the mechanisms linking monocyte activation, PRRs signaling, soluble inflammatory mediators, and markers of monocyte/macrophage activation such as sCD163 to the clinical outcomes remain poorly understood. This understanding of the above immunological mechanisms is crucial to determining the biomarkers of disease progression and possible therapeutic targets. Consequently, the present study investigated the clinical and immunological profiles of laboratory-confirmed CHIKV cases to identify the immune signatures—particularly those involving sCD163 levels, CD16+ monocyte subsets, TLR4/TLR7 expression, cytokines, GFs, and coagulation-related mediators—which may contribute to the exacerbation of inflammation in the acute phase, as well as its persistence in the chronic phase.

## 2. Materials and Methods

### 2.1. Ethics Statement

This study was conducted in accordance with the principles of the Declaration of Helsinki and was approved by the Research Ethics Committee of Plataforma Brasil, Oswaldo Cruz Institute, Oswaldo Cruz Foundation, Ministry of Health, Brazil (CAAE—57221416.0.1001.5248).

### 2.2. Study Population

In 2016 and 2018, suspected cases of arboviral infection in Rio de Janeiro, Brazil, were evaluated at the Hospital Rio Laranjeiras and Hospital dos Plantadores de Cana. Samples were collected during acute, post-acute, and chronic phases. Suspected cases of CF were defined as patients presenting with a sudden onset of fever and severe acute arthralgia, along with an epidemiological link to a laboratory-confirmed case, in accordance with the guidelines from the Brazilian Ministry of Health [20,21]. As an inclusion criterion, patients with a positive molecular test for CHIKV via real-time reverse transcription polymerase chain reaction (RT-PCR), as well as negative results for Zika virus (ZIKV) and dengue virus (DENV), were considered eligible. Conversely, patients who tested positive for DENV using either IgM serology or soluble nonstructural protein 1 (NS1) detection were excluded. CHIKV infection was confirmed by detection of viral RNA using RT-PCR, as previously described [22]. In addition, serological detection of anti-CHIKV IgM and IgG was performed using a capture enzyme-linked immunosorbent assay (ELISA; Euroimmun, Lübeck, Germany).

To comprehensively characterize the clinical, demographic, and disease phase profiles, we enrolled 93 laboratory-confirmed CHIKV cases, defined by positive RT-qPCR results for CHIKV and negative results for DENV and ZIKV. An additional group of ten healthy donors (HD) was included as an uninfected controls. All 93 patients were clinically assessed, and the number of samples included in each immunological assay varied depending on the availability.

Based on the duration of symptoms, patients with CF are classified into three phases according to the Brazilian Ministry of Health: acute (lasting up to 14 days), post-acute (15–90 days), and chronic (>91 days) [21]. Although several patients were lost to follow-up during the chronic phase, it was possible to monitor eight patients at two time points: during the acute/post-acute phase and the chronic phase. Samples from the chronic phase were obtained from patients who were continuously monitored since their initial visit for persistent symptoms, laboratory-confirmed CHIKV infection, as well as negative DENV and ZIKV results. The demographic, clinical, and laboratory data of the patients are presented in Table 1.

### 2.3. Laboratorial Diagnostics

Total RNA was extracted from the plasma samples of patients with suspected CHIKV infection using the QIAamp Viral RNA Mini Kit (Qiagen, Hilden, Germany), according to the manufacturer’s instructions. Molecular tests for CHIKV, DENV, and ZIKV were performed on all samples from the acute, post-acute, and chronic phases [22,23,24]. CHIKV RNA was quantified via RT-qPCR using a standard curve constructed according to the protocol described by Lanciotti et al. [22]. Serological tests were performed on all samples collected for both DENV and CHIKV infections. For DENV, the following assays were used: Panbio Dengue IgM Capture ELISA Kit (Alere™, Brisbane, Australia), Dengue Virus IgM Capture DxSelect™ (Focus Diagnostics, Cypress, CA, USA), and Platelia™ Dengue NS1 Ag ELISA (Bio-Rad Laboratories, Hercules, CA, USA). For CHIKV, the Euroimmun Anti-CHIKV IgM and IgG ELISA Kits (Euroimmun, Lübeck, Germany) were employed. All the tests were performed as per the manufacturer’ instructions. However, the serological diagnosis of ZIKV was not performed due to the lack of a reliable assay with minimal cross-reactivity with other flaviviruses [25].

### 2.4. Blood Samples Collection

Whole blood, serum, and plasma samples were collected from the patients. Approximately 30 mL of blood was drawn into ACD anticoagulant and dry tubes (BD Biosciences, San Jose, CA, USA) for further processing.

### 2.5. Separation of Peripheral Blood Mononuclear Cells

Following the collection of blood samples, peripheral blood mononuclear cells (PBMCs) were isolated using a Ficoll-Histopaque™ gradient (Sigma Aldrich, St. Louis, MO, USA). Briefly, 10 mL blood was carefully layered onto 6 mL Ficoll in centrifuge tubes and centrifuged for 30 min at 400× *g*, with zero brake. Subsequently, the density gradient was separated into layers (sediment > Ficoll > PBMCs > plasma), from which the PBMC layer was carefully collected. The cells were washed twice with RPMI medium (Gibco, Thermo Fisher Scientific, Waltham, MA, USA) and resuspended for viable cell counting using Trypan Blue (LGC Biotechnology, Teddington, UK). Following enumeration, the PBMCs were centrifuged at 400× *g* for 7 min and resuspended in fetal bovine serum (FBS) (Gibco, Thermo Fisher Scientific, Waltham, MA, USA). Thereafter, the cells were aliquoted into a cryopreservation solution composed of 90% FBS and 10% dimethyl sulfoxide (DMSO) (Sigma Aldrich, St. Louis, MO, USA). The PBMCs were cryopreserved in 1 mL cryovials at concentrations of 1 × 10^6^ cells/vial. After suspension in the cryoprotectant solution, the cells were immediately transferred to a controlled-rate freezing container and stored at −80 °C for 24 h to ensure gradual temperature reduction. Subsequently, the cryovials were transferred to liquid nitrogen tanks for long-term preservation. Plasma aliquots of 500 µL were transferred to cryotubes and stored at −80 °C.

### 2.6. Determination of Coagulation, GFs and Estradiol

Circulating levels of TF and TFPI in HD and CHIKV-infected patients were measured using the Human Coagulation Factor II/Tissue Factor ELISA Kit (R&D Systems, Minneapolis, MN, USA) and the Human TFPI ELISA Kit (R&D Systems, Minneapolis, MN, USA), following the manufacturer’s instructions. Similarly, circulating levels of the growth factors EGF, Platelet-derived growth factor (PDGF-BB), and VEGF were quantified in plasma samples collected from HD and CHIKV-infected patients. The assays used for quantification were the Human VEGF Standard TMB ELISA Development Kit (PeproTech, Thermo Fisher Scientific, Cranbury, NJ, USA), the Human PDGF-BB Mini ABTS ELISA Development Kit (PeproTech, USA), and the Human EGF Mini ABTS ELISA Development Kit (PeproTech, USA), all performed according to the manufacturer’s protocols. Serum estradiol (E2) levels were measured using a competitive estradiol assay on the ADVIA Centaur system (Siemens, Munich, Germany) at the Richet laboratory, clinical research, Rio de Janeiro, Brazil, following the manufacturer’s instructions.

### 2.7. Cytokines and sCD163 Determination

Plasma circulating levels of soluble CD163 (sCD163) in HD and CHIKV-infected patients were quantified using ELISA. A commercial Human CD163 DuoSet ELISA kit (R&D Systems, Minneapolis, MN, USA) was used for the same. Additionally, circulating levels of interleukins IL-6, IL-10, IL-17A and Tumor necrosis factor alpha (TNF-α) were measured using the LEGENDplex™ Human CD8/NK Panel (13-plex) (BioLegend, San Diego, CA, USA), as per the manufacturer’s instructions.

### 2.8. Flow Cytometry-Based Characterization of Monocyte Subsets and Surface Markers

As described in the literature, human circulating monocytes are subdivided into distinct subsets based on the expression of the surface markers CD14 and CD16. Approximately 90% of circulating monocytes are classical monocytes characterized by high CD14 expression and absence of CD16 (CD14++CD16− ). The remaining 10% consist of non-classical monocytes (CD14+CD16++), which exhibit higher CD16 and lower CD14 expression, as well as intermediate monocytes (CD14+CD16+) [16]. Further distinction between these subsets can be made based on the differential expression of HLA-DR and CD163. Intermediate monocytes, for instance, express higher levels of HLA-DR than the classical and non-classical subsets in healthy individuals [26,27]. Accordingly, this study classified the monocyte subpopulations based on the combined expression patterns of CD14, CD16, HLA-DR, and CD163. The gating strategy employed to identify these subsets has been illustrated in Supplementary Figure S1A–C using HD samples.

For immunostaining of PBMCs, the following monoclonal antibodies were used: anti-CD14-V500 (BD Biosciences, San Jose, CA, USA), anti-CD16-PECy7 (BioLegend, San Diego, CA, USA), anti-CD163-BV650 (BD Biosciences, San Jose, CA, USA), anti-HLA-DR-PE-Cy5 (R&D Systems, Minneapolis, MN, USA), anti-TLR4-Alexa 488 (eBiocience, San Diego, CA, USA), anti-TLR7-PE (eBiocience, San Diego, CA, USA), and anti-CD3-AF700 (BD Biosciences, San Jose, CA, USA). Additionally, corresponding isotype controls were included. Thawed cells were resuspended in 5 mL of undiluted FBS (Gibco, Thermo Fisher Scientific, Waltham, MA, USA) and centrifuged at 450× *g* for 5 min. Subsequently, the cells were resuspended in supplemented RPMI medium (Gibco, Thermo Fisher Scientific) and counted using a TC20™ Automated Cell Counter (Bio-Rad Laboratories, Hercules, CA, USA). The cell density was adjusted to 5 × 10^5^ cells/well. Following a second centrifugation step, the cells were stained with the Live/Dead™ viability dye-Pacific blue (Molecular Probes, Thermo Fisher Scientific, Waltham, MA, USA) according to the manufacturer’s instructions. Extracellular staining was performed first. Briefly, the cells were incubated for 20 min in a blocking buffer consisting of phosphate-buffered saline (PBS), along with 1% bovine serum albumin (BSA) and 5% heat-inactivated human plasma. Thereafter, the cells were incubated for 30 min at 4 °C with a cocktail of monoclonal antibodies targeting surface markers, following which they were washed and fixed in 4% paraformaldehyde diluted in PBS for 10 min at 4 °C. Subsequently, the cells were permeabilized and subjected to an additional blocking step. Intracellular staining was performed by incubating the cells with monoclonal antibodies for 30 min, followed by a final fixation step with 2% PFA. Finally, samples were resuspended in PBS and transferred into cytometry tubes. Data acquisition was performed using a BD FACSAria IIu flow cytometer (BD Biosciences, San Jose, CA, USA). Data analysis was performed using the FlowJo software version 10 (Tree Star Inc., Ashland, OR, USA).

### 2.9. Statistical Analysis

Statistical analyses were performed using the GraphPad Prism software (GraphPad Software Inc., San Diego, CA, USA; version 10). To compare the differences between the study groups, the non-parametric Kruskal–Wallis test followed by Dunn’s multiple comparisons post hoc test was applied. For unpaired comparisons, the Mann–Whitney U test was used, whereas the Wilcoxon matched-pairs signed-rank test was used for paired analyses. Additionally, the Chi-square test and/or Fisher’s exact test were used to evaluate associations between categorical variables. To explore the potential predictors of joint-related symptoms, multivariate regression analyses were conducted, adjusting for possible confounders such as age, sex, and disease phase. Furthermore, principal component analysis (PCA) was performed to reduce the dimensionality and identify monocyte subpopulation patterns associated with different disease phases. The Kaiser–Meyer–Olkin measure of sampling adequacy and Bartlett’s test of sphericity were used to evaluate the suitability of the data for PCA. Moreover, varimax rotation was applied to maximize the variance of factor loadings across components. Individual component scores were subsequently normalized to a scale of 0 -1, and the differences between patient groups across disease phases were assessed. A PCA plot was generated using the factoextra package in the R programming environment. Differences were considered statistically significant at *p* < 0.05.

## 3. Results

### 3.1. Baseline Characteristics and Laboratorial Parameters of Patients with CF

Blood samples were collected at Hospital Rio Laranjeiras and Hospital dos Plantadores de Cana. Suspected CF cases were defined as patients presenting with sudden onset fever greater than 38.5 °C accompanied by severe acute arthralgia, with no other apparent cause [1]. Table 1 summarizes the demographic, clinical, and laboratory characteristics of the 93 patients. The patients were classified into acute (≤14 days), post-acute (15–90 days), and chronic (>91 days) phases, based on the Brazilian Ministry of Health guidelines [21]. All CHIKV cases were attributed to the East–Central–South African (ECSA) lineage [20,28].

With regard to the demographic characteristics, the median age of patients in the acute phase was 45 years (range: 18–76 years), wherein 52 (60%) patients were female. In the post-acute phase, the median age was 56 years (range: 34–66 years), with two female patients (28%). Additionally, the median time from symptom onset to blood collection was 5.0 days (IQR: 3–7 days) during the acute phase and 20 days (IQR: 15–38 days) during the post-acute phase (Table 1). The median viral loads measured in samples during the acute and post-acute phases were 4.7 and 3.5, respectively.

**Table 1:** Demographic, clinical, and laboratorial parameters of CF patients during the time course of the disease.

	Acute *n* = 86	Post-Acute *n* = 7	Chronic *n* = 8
Age (years) *	45 (18–76)	56 (34–66)	64 (46–73)
Female sex; n (%)	52 (60)	2 (28)	4 (50)
Days post-symptom onset ^1^†	5.0 (3–7)	20 (15–38)	91 (91–110)
**Laboratorial Diagnosis**			
RT-PCR	86 (100)	7 (100)	7 (87.5)
Viral load Log copies/mL	4.7 (3.2–6.1)	3.5 (2.8–6.4)	4.1 (3.7–4.7) †
Anti-CHIKV antibodies			
IgM (%) ^2^	40 (40)	7 (100)	8 (100)
IgG (%)	10 (11)	7 (100)	8 (100)
**Clinical data during the time course of the disease, n (%)**			
Fever	78 (90.6)	0 (0)	0 (0)
Temperature, (°C) *	38.9 (38.2–39.0)	-	-
Arthralgia	80 (93.0)	7 (100)	8 (100)
Myalgia	69 (80.2)	4 (57.1)	0 (0)
Prostration	64 (74.4)	4 (57.1)	0 (0)
Headache	63 (73.2)	3 (42.8)	0 (0)
Low back pain	52 (60.4)	5 (71.4)	0 (0)
Loss of appetite	49 (56.9)	4 (57.1)	0 (0)
Edema	47(54.6)	4 (57.1)	4 (50.0)
Exanthem	47 (54.6)	3 (42.8)	0 (0)
Nausea	41 (47.6)	3 (42.8)	0 (0)
Retro-orbital pain	40 (46.5)	2 (28.5)	0 (0)
Pruritus	37 (43.0)	4 (57.1)	0 (0)
Arthritis	27 (31.3)	2 (28.5)	4 (50.0)
Vertigo	25 (29.1)	2 (28.5)	0 (0)
Conjunctival hyperemia	15 (17.4)	0 (0)	0 (0)
Vomiting	14 (16.2)	0 (0)	0 (0)
Abdominal pain	13 (15.1)	0 (0)	0 (0)
Paresthesia	8 (9.30)	1 (14.2)	0 (0)
Diarrhea	7 (8.13)	2 (28.5)	0 (0)
Cough	7 (8.13)	0 (0)	0 (0)
Runny noise	7 (8.13)	0 (0)	0 (0)
Epistaxis	1 (1.16)	0 (0)	0 (0)
Facial and limb paralysis	1 (1.16)	0 (0)	0 (0)
**Laboratorial parameters**			
Hematocrit †	36.2 (34.4–39.1)	36.8 (36.6–40.1)	41.5 (36.8–43.1)
Platelets × 10^3^ count/mm^3^	200 (154–252)	238 (172–274)	271 (195–385) ^3^
Leukocytes count/mm^3^	5300 (3775–6125)	4765 (3708–6475)	7925 (6123–10,165)
Lymphocytes count/mm^3^	1220 (856–1813)	1169 (1060–1531)	1740 (1325–2448)
Monocytes count/mm^3^	375 (300–564)	359 (248–477)	520 (352–587)
Neutrophils count/mm^3^	3188 (2135–4214)	2891 (1427–4484)	4740 (3975–6638) ^2^

(*) data are expressed as median (min–max); ^1^ Days from disease onset until the interview. † data are expressed as median interquartile range. ^2^ number (%). ^3^ Statistical analysis by one-way ANOVA followed by Kruskal–Wallis and Dunn’s multiple comparisons test or Mann–Whitney *t* test.

The patients exhibited a broad spectrum of articular as well as systemic symptoms. Among the 93 participants, musculoskeletal pain (myalgia associated with arthralgia) was consistently reported in all phases. Most patients were febrile during the acute phase (78/86, 90.6%), with a median temperature of 38.9 °C. Arthralgia was the most frequent symptom (acute: 80/86, 93.0%; post-acute: 7/7, 100%), followed by myalgia (acute: 69/86, 80.2%; post-acute: 4/7, 57.1%), prostration (acute: 64/86, 74.4%; post-acute: 4/7, 57.1%), headache (acute: 63/86, 73.2%; post-acute: 3/7, 42.8%), lower back pain (acute: 52/86, 60.4%; post-acute: 5/7, 71.4%), and edema (acute: 47/86, 54.6%; post-acute: 4/7, 57.1%).

Gastrointestinal symptoms were also reported in both the acute as well as post-acute phases. Loss of appetite was the most common symptom (acute: 49/86, 56.9%; post-acute: 4/7, 57.1%), followed by nausea (acute: 41/86, 47.6%; post-acute: 3/7, 42.8%). Less frequent manifestations included vomiting (acute: 14/86, 16.2%; post-acute: 0/7, 0%), abdominal pain (acute: 13/86, 15.1%; post-acute: 0/7, 0%), and diarrhea (acute: 7/86, 8.1%; post-acute: 2/7, 28.5%).

The most prevalent cutaneous manifestations during the acute phase were exanthema (acute: 47/86, 54.6%; post-acute: 3/7, 42.8%) and pruritus (acute: 37/86, 43.0%; post-acute: 4/7, 57.1%). Vertigo was reported in 27 patients (acute: 25/86, 29.1%; post-acute: 2/7, 28.5%). Additional manifestations included conjunctival hyperemia (acute: 15/86, 17.4%), paresthesia (acute: 8/86, 9.3%; post-acute: 1/7, 14.2%), as well as cough and rhinorrhea (acute: 7/86, 8.1%). One patient developed neurological complications suspected to be Guillain-Barré syndrome and required hospitalization. Furthermore, epistaxis and facial or limb paralysis were reported in a single patient each. No deaths occurred during the study period.

A total of 42 patients presented with comorbidities. Hypertension was the most prevalent condition (21/42; 50%), followed by diabetes (7/42; 16.6%), allergic rhinitis (3/42; 7.1%), and asthma (7/42; 16.6%). Two (2/42, 4.8%) patients had gastritis. Dementia, Crohn’s disease, cardiovascular disease, and hypothyroidism were reported in one patient each.

In the chronic phase, four out of eight patients (50.0%) were female, with a median age of 64 years. Seven of eight chronic patients (87%) tested positive for chikungunya by RT-PCR, with a median viral load of 4.1 log copies/mL (IQR: 3.7–4.7), while all tested negative for dengue and zika by RT-PCR. Additionally, all patients with chronic disease presented with detectable anti-CHIKV IgM and IgG antibodies. The median time from symptom onset to blood collection was 91 days (IQR: 91–110 days), wherein the most common clinical manifestations included persistent arthralgia, edema, and arthritis. Moreover, the primary comorbidities were hypertension (2/8, 25.0%), diabetes mellitus (1/8, 12.5%), gastritis (1/8, 12.5%), allergic rhinitis (1/8, 12.5%), and cardiovascular disease (1/8, 12.5%). There were no statistically significant differences in the viremia levels between the acute, post-acute, and chronic phases.

Furthermore, the laboratory data of 43 patients were analyzed, whereby the hematological parameters revealed only mild alterations. Platelet counts were decreased in patients during the acute phase (median 200 × 10^3^/mm^3^, IQR 154–252/mm^3^) compared to those in the chronic phase (median 271 × 10^3^/mm^3^, IQR 195–385/mm^3^, *p* = 0.04). Leukocyte counts were also lower in the acute phase (median 5300 × 10^3^/mm^3^, IQR 3775–6125/mm^3^) compared to the chronic phase (median 7925 × 10^3^/mm^3^, IQR 6123–10,165/mm^3^, *p* = 0.0086). A similar pattern was observed for neutrophil counts, with reduced circulating levels in acute phase of infection (median 3188 × 10^3^/mm^3^, IQR 2135–4214/mm^3^) relative to the chronic phase (median 4740 × 10^3^/mm^3^, IQR 3975–6638/mm^3^, *p* = 0.0076). Despite these findings, thrombocytopenia was observed in eight patients, leukopenia in 15, lymphopenia in seven, neutropenia in seven, and monocytopenia in one patient.

The clinical characteristics of the confirmed cases were generally similar between males and females, except for the frequency of edema, which was significantly higher in females than in males (66.6% vs. 38.4%, *p* = 0.0029) (Table 2).

### 3.2. Dynamic Changes of Monocytes in CF Patients During Acute and Chronic Phases of CHIKV Infection

**Figure 1:**
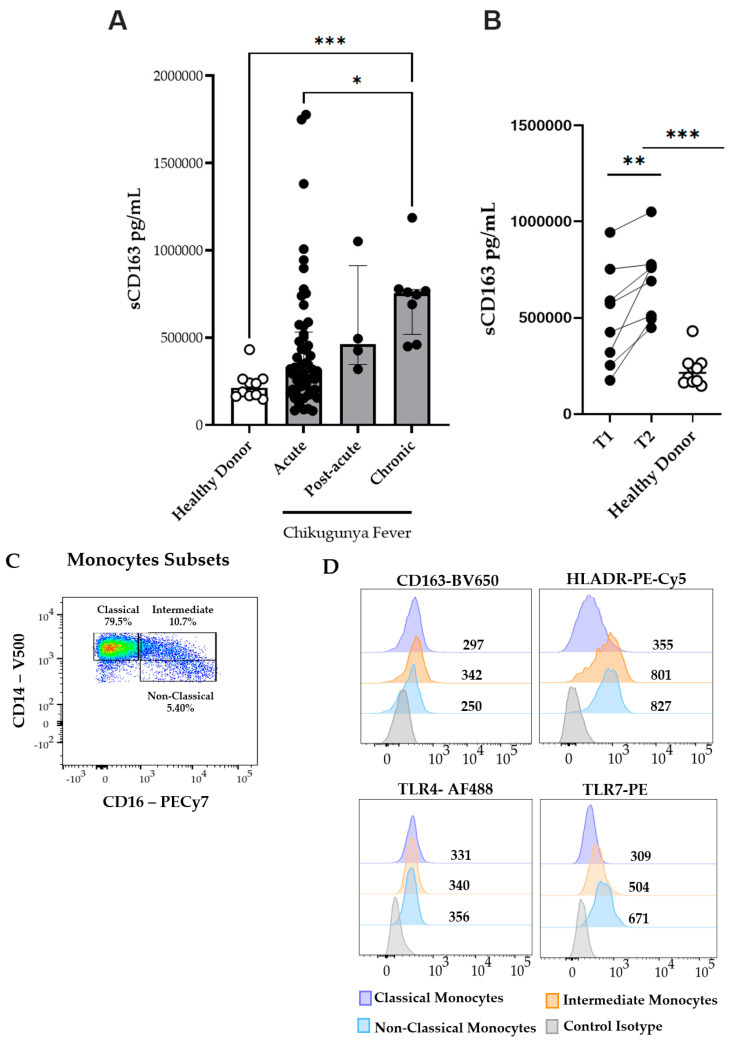

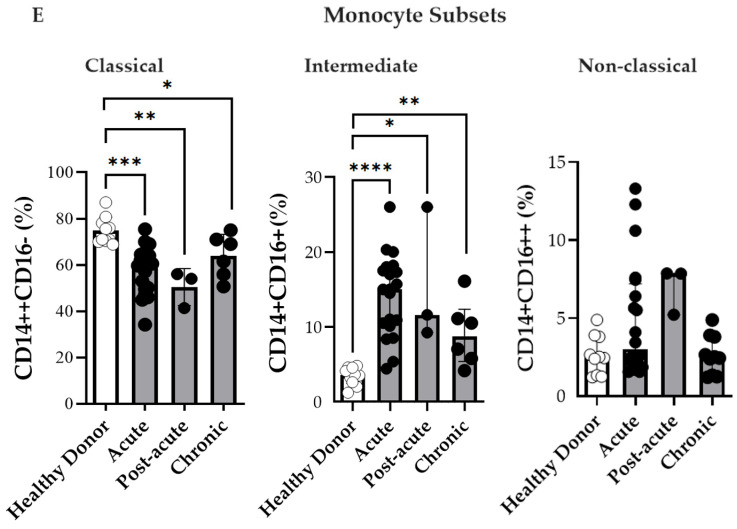
Circulating plasma levels of soluble CD163 (sCD163) and monocyte subsets during the acute (n = 53), post-acute (n = 4), and chronic (n = 8) phases of CF. (**A**) Plasma levels of soluble CD163 (sCD163) in CF patients and healthy donors (HD). (**B**) Paired comparison of sCD163 plasma levels. Time Point 1 (T1) represents the first sample collected during the acute or post-acute phase, and Time Point 2 (T2) represents the second sample collected during the chronic phase. (**C**) Representative dot plot from a CHIKV-infected patient in the acute phase showing monocyte subsets. (**D**) Representative histograms of CD163, HLA-DR, TLR4, and TLR7 expression on classical, intermediate, and non-classical monocytes. (**E**) Percentages of circulating classical (CD14++CD16−), intermediate (CD14+CD16+), and non-classical (CD14+CD16++) monocytes analyzed in CF patients during the acute (n = 20), post-acute (n = 3), and chronic (n = 6) phases, and in healthy donors (HD, n = 10). Data are presented as medians with interquartile ranges (IQR). Statistical analysis: one-way ANOVA followed by Kruskal–Wallis and Dunn’s multiple comparisons test; Wilcoxon signed-rank test for paired comparisons. * *p* < 0.05; ** *p* < 0.01; *** *p* < 0.001; **** *p* < 0.0001.

Monocyte activation plays a central role in the pathogenesis and progression of chikungunya virus infection, as evidenced by elevated levels of activation markers such as soluble CD163 [13,29]. CD163 is a membrane-bound scavenger receptor that is cleaved and released into circulation upon monocyte/macrophage activation [30]. In addition, CD163 is widely recognized as a biomarker associated with immune regulation and inflammation, particularly in M2-polarized macrophages, which are involved in anti-inflammatory processes and tissue repair [31]. Therefore, we quantified soluble CD163 (sCD163) levels in the plasma. Our data demonstrated that sCD163 concentrations were significantly elevated during the acute phase of infection compared to HD. Moreover, sCD163 levels showed a significant increase in the chronic phase compared to the acute phase, indicating a progressive increase in soluble CD163 levels throughout the course of CHIKV infection (Figure 1A). We examined paired samples to investigate longitudinal changes in sCD163 expression. As shown in Figure 1B, sCD163 levels were significantly higher in the chronic phase than in the acute or post-acute phases, suggesting an association between disease progression and sustained monocyte/macrophage activation.

To further understand the cellular mechanisms underlying the observed increase in sCD163 levels, we investigated the frequency and activation profiles of monocyte subsets during CHIKV infection. A representative flow cytometry plot from a patient in the acute phase is depicted in Figure 1C, illustrating a marked increase in intermediate (CD14+CD16+) and non-classical (CD14+CD16++) monocyte subsets. Compared to HD, CHIKV-infected patients exhibited lower frequencies of classical monocytes (CD14++CD16− ) during the acute and post-acute phases, as illustrated in Figure 1E. Conversely, infected patients demonstrated higher frequencies of intermediate (CD14+CD16+) monocytes; although non-classical (CD14+CD16++) monocytes were also elevated, this increase was not statistically significant. Thus, the acute and post-acute stages of CHIKV infection are characterized by the expansion of intermediate and non-classical monocyte subsets. Owing to low sample viability, only six chronic-phase samples were available for analysis. These changes highlighted a shift in the monocyte subset composition associated with CHIKV infection.

### 3.3. Monocyte Activation During Chikungunya Fever

**Figure 2:**
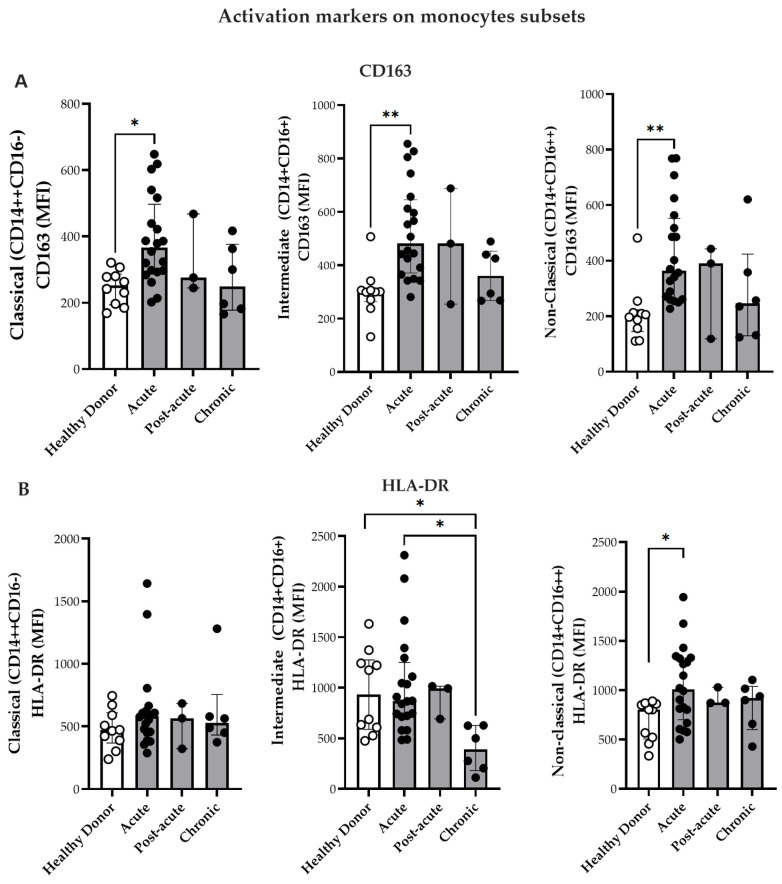

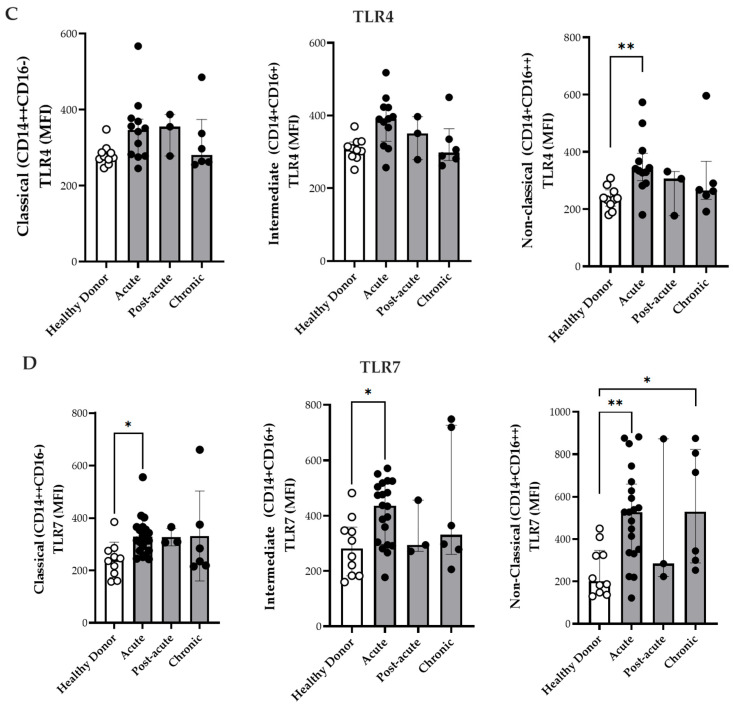
Expression of activation markers on monocyte subsets. (**A**) Comparison of CD163 expression on monocyte subsets from CF patients during acute (n = 20), post-acute (n = 3), and chronic phases (n = 6) in HD (n = 10). (**B**) Comparison of HLA-DR expression on monocyte subsets from CF patients during acute (n = 20), post-acute (n = 3), and chronic phases (n = 6) and in HD (n = 10). (**C**) Comparison of TLR4 expression on monocyte subsets from patients in the acute (n = 12), post-acute (n = 3) and chronic (n = 6) phases, and in HD (n = 10). (**D**) Comparison of TLR7 expression on monocyte subsets from patients in the acute (n = 20). Post-acute (n = 3) and chronic phases (n = 6) phases, and in HD (n = 10). Mean fluorescence intensity (MFI) of labeled cells was determined by flow cytometry. Data are presented as medians with interquartile ranges (IQRs). Statistical analysis: one-way ANOVA followed by Kruskal–Wallis test and Dunn’s multiple comparisons test and using the Mann–Whitney test. * *p* < 0.05; ** *p* < 0.01.

Thereafter, monocyte activation was assessed by evaluating the expression of cell surface markers CD163 and HLA-DR. HLA-DR is a critical molecule expressed on professional antigen-presenting cells that express viral peptides and subsequently initiate antiviral immune responses [32]. In Figure 1D, representative histograms illustrate the expression levels of the activation markers HLA-DR, CD163, TLR4, and TLR7 in the monocyte subsets. After measuring the sCD163 levels, the expression of CD163—a transmembrane scavenger receptor primarily expressed on monocytes and macrophages—was examined on the cell surface. As depicted in Figure 2A, all the monocyte subsets exhibited elevated CD163 expression during the acute phase of CHIKV infection compared to that in HD.

As illustrated in Figure 2B, intermediate monocytes from HD and CHIKV-infected patients exhibited higher HLA-DR expression during the acute phase than those patients from the chronic phase. Moreover, non-classical monocytes also exhibited increased HLA-DR mean fluorescence intensity (MFI) during the acute phase relative to HD (Figure 2B). In contrast, no significant differences were observed in HLA-DR expression in the classical monocytes.

CHIKV induces innate immune responses through Toll-like receptors (TLRs)—particularly TLR3, TLR7, and TLR8—which are involved in viral RNA sensing [33,34]. Although changes in the expression have been described in patients with viremic CHIKV [33], the differential expression of TLRs among monocyte subsets has not yet been explored. Therefore, the present study investigated the expression of TLR4 and TLR7 in the monocyte subsets (Figure 2C,D). TLR4 MFI expression was significantly elevated in the non-classical monocytes during the acute phase compared with that in HD (Figure 2C). Additionally, the classical and intermediate monocytes exhibited higher TLR7 MFI in the acute phase than in HD group (Figure 2D). Non-classical monocytes demonstrated increased TLR7 expression during both the acute and chronic phases compared to the HD group (Figure 2D). Paired analyses of monocyte subsets and activation markers (CD163, HLA-DR, TLR4, and TLR7) exhibited no significant differences between time point 1 (T1, acute phase/post-acute) and time point 2 (T2, chronic phase). The only significant change was a decrease in CD163 expression on non-classical monocytes [(T1 396 (359–579) vs. T2 246 (122–423) *p* = 0.032)]. This corresponded to the plasma sCD163 data, thereby suggesting the proteolytic cleavage and ongoing activation of these cells.

### 3.4. PCA of Monocytes Subsets

**Figure 3:**
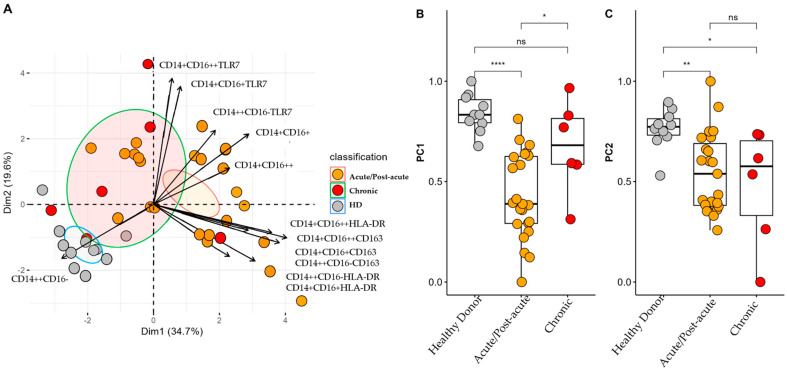
Profiles of Monocyte Subsets in Patients with CF during the Acute/Post-Acute and Chronic Phases of Infection. (**A**) Principal component analysis (PCA) biplot showing two principal components that together account for 54.3% of the cumulative variance. Individual scores for each principal component (PC) are represented as colored points, with ellipses indicating the 95% confidence interval for each group. Variable loadings are represented as arrows; the length of each arrow indicates the contribution of the corresponding variable to each principal component. (**B**,**C**) Comparison of individual scores for PC1 and PC2. * *p* < 0.05; ** *p* < 0.01; **** *p* < 0.0001. ns: not significant.

Monocyte subpopulation analysis was performed on 39 samples (20 from patients in the acute phase, 3 in the post-acute, phase and 6 in the chronic phase) from patients with CHIKV and ten samples from HD. The Kaiser–Meyer–Olkin test yielded a value of 0.64, indicating moderate sampling adequacy, whereas the results of the Bartlett’s test of sphericity was also significant (*p* < 0.001). Furthermore, the determinant of the correlation matrix was <0.001, confirming its suitability for PCA. As depicted in Figure 3A, PCA revealed three distinct clusters corresponding to HD, acute/post-acute phase, and chronic phase patients. Principal components 1 and 2 (PC1 and PC2) explained 34.7% and 19.6% of the total variance, accounting for a cumulative 54.3%. The monocyte subpopulations CD14+CD16+ and CD14+CD16++ expressing TLR7 were predominantly associated with the chronic phase, which suggested persistent TLR7-mediated activation. In contrast, the CD14++CD16-HLA-DR and CD14+CD16+HLA-DR subsets were more prominent in the acute/post-acute phase, indicating different activation profiles.

Higher scores on PC1 revealed that HD tended to cluster closely together, indicating less variability and a more stable monocyte profile (Figure 3B). In contrast, patients in the acute/post-acute phase displayed marked alterations, likely driven by the expansion of the intermediate monocytes (CD14+CD16+) and a reduction in the classical monocytes (CD14++CD16−), along with a partial return to baseline observed in the chronic phase. In PC2, HD again demonstrated low variability, but patients in the acute/post-acute phase presented significantly lower values. This potentially reflected the increased activation of monocytes expressing TLR7, particularly in the intermediate and non-classical subsets. However, patients in the chronic phase displayed greater heterogeneity, suggesting that persistent immune activation may occur in some individuals.

### 3.5. Circulating Levels of Inflammatory, Coagulation, and Immune Activation Markers During CHIKV Infection

#### 3.5.1. Pro-Inflammatory and Anti-Inflammatory Cytokines

**Figure 4:**
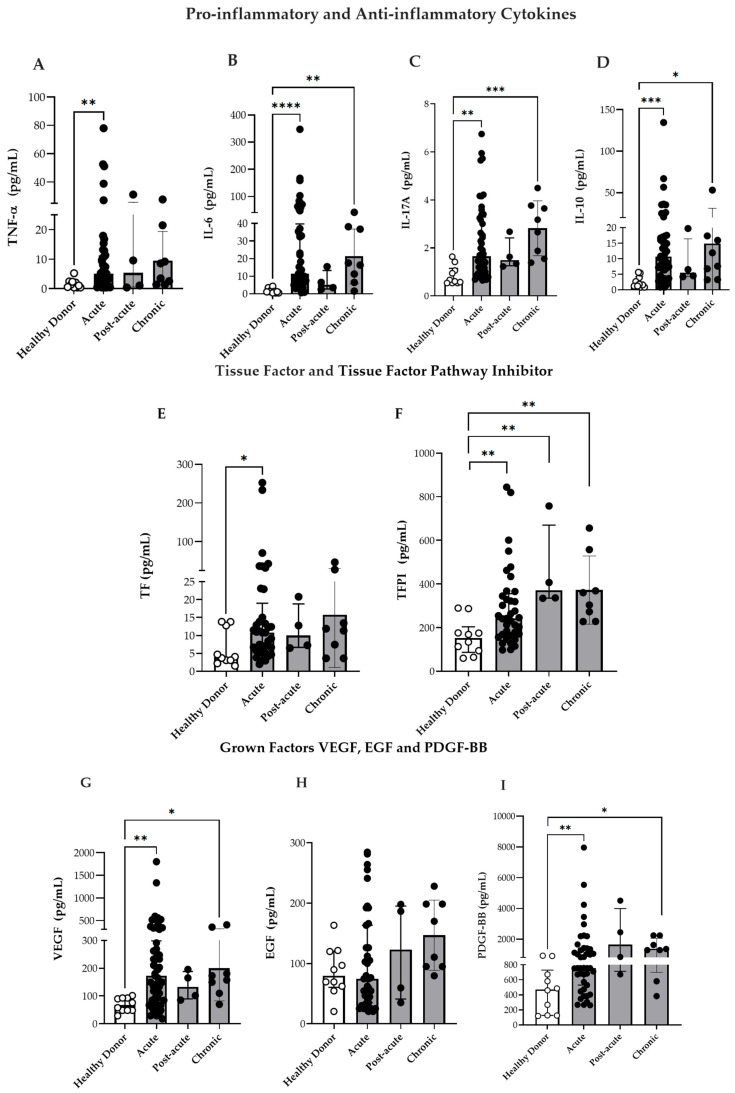
Circulating markers of inflammation and vascular dysfunction. (**A**–**D**) Circulating levels of IL-6, TNF-α, IL-17A, and IL-10 in patients with CF during the acute (n = 46), post-acute (n = 4), chronic (n = 8) phases of infection, and in HD (n = 10). (**E**,**F**) Circulating levels of TF and TFPI in CF patients during the acute (n = 37), post-acute (n = 4), and chronic (n = 8) phases, and in HD (n = 10). (**G**) Circulating levels of VEGF in CF patients during the acute (n = 53), post-acute (n = 4) and chronic phases (n = 8), and in HD (n = 10). (**H**) Circulating levels of EGF in CF patients during the acute (n = 39), post-acute (n = 4), chronic phases (n = 8), and in HD (n = 10). (**I**) Circulating levels of PDGF-BB in CF patients during the acute (n = 43), post-acute (n = 4), chronic phases (n = 8), and in HD (n = 10). Data are presented as medians with interquartile ranges (IQR). * *p* < 0.05, ** *p* < 0.01, *** *p* < 0.001, **** *p* < 0.0001; one-way ANOVA followed by Kruskal–Wallis test and Dunn’s multiple comparisons test and using the Mann–Whitney test.

Taking into consideration that CHIKV infection induces a highly differentiated phenotype among monocyte subsets and activates the TLR4 and TLR7 pathways, this study also measured the plasma concentrations of pro- and anti-inflammatory cytokines as well as coagulation and GFs. The levels of IL-6, IL-17A, and IL-10 were elevated during the acute phase and remained persistently high during the chronic phase (Figure 4B–D), which was in agreement with the findings of previous studies [35,36]. Furthermore, the circulating TNF-α levels were elevated during the acute phase compared to that in HD (Figure 4A). In general, patients had increased concentrations of IL-17A in the acute phase, which remained higher in the chronic phase, although no differences were found between the paired samples (acute/post-acute vs. chronic). Moreover, the IL-10 plasma levels were higher in the acute phase, and some patients maintained elevated levels during the chronic phase. However, analysis of paired samples revealed significantly decreased IL-10 concentrations in the chronic phase compared to the acute/post-acute [median 16.8 (7–28) pg/mL in the acute vs. 6.6 (3.9–12.9) pg/mL in the chronic phase; *p* = 0.0098].

#### 3.5.2. Coagulation Factors and GFs

It is well established that monocytes are a major source of TF, the primary initiator of the coagulation cascade [37,38]. During vascular injury, TF is exposed to circulating blood, where it binds to Factor VII, and results in its activation (Factor VIIa). This subsequently activates Factors IX and X, ultimately resulting in fibrin clot formation [37]. TFPI—which is primarily produced by endothelial cells—counteracts this process by binding to Factor Xa and inhibiting the TF–Factor VIIa complex, thus playing a critical role in the regulation of coagulation [39].

Previous studies have highlighted the role of TF as a key mediator linking hemo-stasis and inflammation during arboviral infections [40,41]. Furthermore, dysregulation of coagulation factors has been implicated in various chronic inflammatory diseases, including rheumatoid arthritis (RA) [42,43]. In this context, our goal was to verify TF and TFPI levels during CHIKV infection. As depicted in Figure 4E,F, both TF as well as TFPI levels were elevated during the acute phase, and TFPI was maintained at higher levels in the post-acute and chronic phases than in HD. However, the TF/TFPI or TFPI/TF ratios were not significantly increased in CHIKV infection: TFPI/TF ratio [HD 28 (19–60), acute 27 (10–44), post-acute 52 (23–62) and chronic 29 (12–70)] or TF/TFPI ratio [HD 0.030 (0.016–0.041), acute 0.036 (0.022–0.098), post-acute 0.019 (0.016–0.052) and chronic 0.034 (0.014–0.081)].

GFs—such as VEGF, PDGF, and EGF play important roles in modulating circulating blood monocytes by influencing their recruitment, differentiation, and responsiveness to inflammatory stimuli [44]. Taking in to consideration the alterations observed in monocyte subpopulations during CHIKV infection, this study assessed the plasma concentrations of PDGF-BB, EGF, and VEGF in the infected patients. Consequently, the VEGF and PDGF-BB levels were consistently elevated in the acute, and chronic phases compared to those in HD (Figure 4G,I). Although EGF levels were higher in CHIKV-infected individuals, no statistically significant differences were observed (Figure 4H). These findings suggest that CHIKV infection is associated with both inflammatory and vascular dysregulation, which likely contribute to the disease pathophysiology.

#### 3.5.3. Elevated TLR7+Intermediate Monocytes and Vascular Markers in Patients with CF, Along with Articular Manifestations

**Figure 5:**
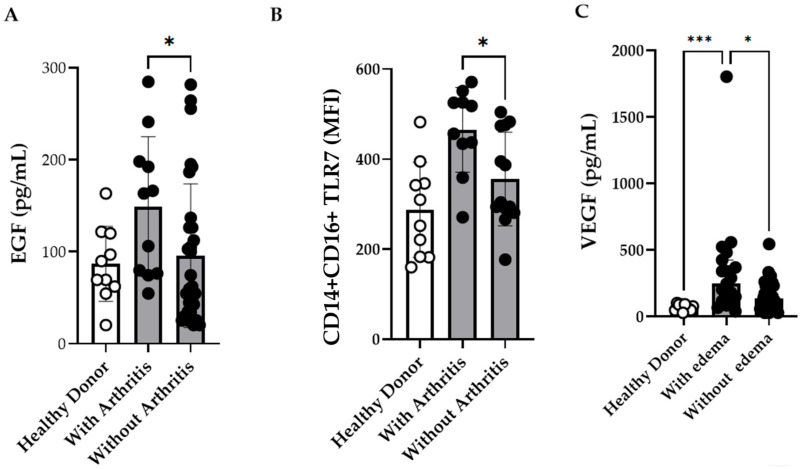
Intermediate CD14+CD16+ Monocytes Expressing TLR7 and Plasma Concentrations of EGF, and VEGF in Chikungunya Fever (CF) Patients with Arthritis and Edema. (**A**) Plasma concentrations of EGF in CF patients with arthritis (*n* = 11) and without arthritis (*n* = 30) (**B**) Comparison of TLR7 expression on intermediate monocytes from CF patients with arthritis (*n* = 10) and without arthritis (*n* = 13). (**C**) Plasma concentrations of VEGF in CF patients with edema (*n* = 17) and without edema (*n* = 38). Healthy donors (HD) (*n* = 10) were included as controls. Data are presented as medians with interquartile ranges (IQR; Kruskal–Wallis test followed by Dunn’s multiple comparisons test and using the Mann–Whitney test. *p*-values < 0.05 were considered significant). * *p* ≤ 0.05 *** *p* ≤ 0.001.

Previous studies have investigated the complex interactions between cytokines and immune responses in patients with severe CF [2,35]. As our cohort included only one severe case, we focused on the association between immunological markers and joint-related symptoms such as edema and arthritis. EGF levels were observed to be elevated in patients with arthritis (Figure 5A; Supplementary Table S4), whereas patients presenting with edema had significantly higher VEGF concentrations (Figure 5C; Supplementary Table S3). Similarly, patients with arthritis exhibited significantly higher TLR7 MFI in intermediate monocytes than those without arthritis (Figure 5B). However, owing to the limited sample size, these associations were not retained in the multivariate regression analyses.

Subsequently, the Spearman’s correlations between monocyte subpopulations and all measured inflammatory mediators were also analyzed. In particular, the cytokines IL-6, IL-10, TNF-α, and IL-17A were positively correlated. Furthermore, IL-10 was positively correlated with intermediate monocytes, whereas CD163 expression on these cells was inversely correlated with IL-10 levels. Additionally, a positive correlation between EGF and TNF-α was observed (Supplementary Table S1).

#### 3.5.4. Sex as a Biological Variable in CHIKV Infection

Women may be more prone to developing chronic arthralgia even after the resolution of acute infection [45,46]. TLR7 has received significant attention in relation to understanding the mechanisms underlying the female predisposition to autoimmune diseases, as it plays a critical role in the immune response and is encoded on the X chromosome [47]. However, the mechanisms underlying sex-linked differences in immune responses remain unclear [48]. To address this, the present study examined sex-related variations in monocyte subsets and the plasma concentrations of inflammatory mediators. There were no statistically significant differences in the distribution of monocyte subsets or TLR7 expression between the sexes (Supplementary Table S2). Similarly, no significant differences were observed in the circulating levels of inflammatory cytokines or GFs. However, a significant reduction in plasma TFPI concentrations was observed in females compared with males [Female: median 231 (146–319) vs. Male: 325 (205–528) IQR, *p* = 0.0347] (Supplementary Table S2). Upon stratification by age, women who were less than 50 years-old exhibited significantly lower TFPI concentrations than those aged > 50 years [137 (11–171), vs. 269 (224–340)]. Serum estradiol levels were also measured, confirming that women aged < 49 years had significantly higher estradiol concentrations than their counterparts over 50 years [47.4 (22–114) vs. 23 (20.3–45) pg/mL]. Finally, subgroup analyses did not exhibit any statistically significant differences in the immunological parameters between young and elderly patients, or between those with and without comorbidities.

## 4. Discussion

CHIKV has emerged as a significant public health concern owing to its widespread transmission and the clinical complexity of its manifestations. It is transmitted by mosquito bites, and the infection typically presents with fever and severe joint pain. These symptoms may persist long after the acute phase, leading to chronic arthralgia and disability [20,28,49]. This study investigated the clinical manifestations and host immune responses in patients who were naturally infected with CHIKV during the 2016 and 2018 outbreaks in Rio de Janeiro, Brazil. Consistent with previous reports, the predominant symptoms included fever, arthralgia, and myalgia, along with extra-articular features such as headache and exanthema [45,49]. Furthermore, women experienced symptoms such as edema much more frequently than men. This observed pattern may also be influenced by sociocultural factors, such as women’s proclivity for healthcare-seeking behaviors. Our results align with previous studies reporting greater intensity and prolonged duration of joint and systemic symptoms in women infected with CHIKV [50,51]. Moreover, women are known to mount stronger immune responses than men, which can confer enhanced protection against infections and vaccination, but is also associated with a higher incidence of autoimmune diseases [52].

Following TLR activation, intermediate monocytes—which are powerful antigen-presenting cells—produce cytokines such as TNF- α, IL-6, and IL-1 β. They expand during bacterial and viral infections and several chronic illnesses, including RA [53]. Previous research by our group and others has demonstrated that patients with dengue have increased frequency of intermediate monocytes [54,55]. During CF, the distribution of monocyte subsets also changed significantly during the acute phase of infection. Additionally, the number of classical monocytes (CD14++CD16−) decreased significantly, whereas the number of intermediate (CD14+CD16+) and non-classical (CD14+CD16++) monocytes increased. These findings are consistent with those reported in the literature. An immunological profile study of pediatric patients with CHIKV infection reported an increase in the number of CD14+CD16+ intermediate monocytes during the acute phase of infection [29]. Furthermore, within three days post-infection, monocytes in experimental CHIKV infection models in cynomolgus monkeys exhibit increased expression of CD14 and CD16, suggesting that these cells were activated [56]. In addition, studies have suggested that intermediate monocytes may have both protective and pathogenic functions during alphaviral infections. High levels of the CHIKV envelope protein were found in monocytes, and intermediate monocytes were found to be important contributors to the inflammatory response in infected children [29]. Moreover, ILy6C^+^ intermediate monocytes in the skin have been shown to facilitate viral dissemination to lymph nodes and beyond in murine model [57]. Haist et al. demonstrated that intermediate monocytes are rapidly recruited to the infection site, where they produce substantial amounts of type I interferons, which are critical for controlling viral replication and resolving infections [58]. Furthermore, a recent study demonstrated that CD14+CD16+ monocytes may facilitate CHIKV invasion of the central nervous system (CNS). When infected in vitro, these monocytes can cross the blood–brain barrier, suggesting their potential to transport viruses to the brain [2]. Despite the limited sample size, our findings support the role of intermediate monocytes in CHIKV infection, especially during the acute phase, with some elevation persisting during the chronic phase.

Despite the lack of statistical significance in the frequency of CD14+CD16++ non-classical monocytes in patients with CF, the observed upregulation of HLA-DR expression during the acute phase suggested that these cells were functionally activated [32]. Recently the immunogenic potential of CHIKV through HLA class II molecules, particularly HLA-DR was reported. The study highlighted that 12 viral ligands from the CHIKV polyprotein naturally presented by different HLA-DR, -DP, and HLA class II responses were more significant than those against the vector used for vaccine development, indicating the potential of using HLA-DR in vaccine strategies to enhance CHIKV immunity [59]. Infection of C20 human microglial cells with CHIKV led to significant apoptosis and altered the expression of cell surface markers, including a notable decrease in CD14 and upregulation of HLA-DR expression [60]. Although there was no significant difference in the frequency of CD14+CD16++ cells during the acute phase, Michlmayr et al. demonstrated a significant correlation between the frequency of these cells and the anti-CHIKV antibody titer [29]. Increased CD14 and CD16 expressions in monocytes has been observed in CHIKV-infected cynomolgus macaques. However, this study did not determine whether there was an expansion of non-classical monocytes (CD14+CD16++) [56]. Furthermore, in vitro studies have shown that CHIKV can directly infect human monocytes, resulting in phenotypic changes in monocytes; however, specific subpopulations of monocytes have not been evaluated [14].

HLA-DR is upregulated in non-classical monocytes during the acute phase, whereas a significant reduction in HLA-DR expression is observed in intermediate monocytes during the chronic phase. This pattern is consistent with our previous observations in severe dengue cases [54] and with findings from experimental CHIKV models [56], although its interpretation requires caution because of the limited number of samples analyzed. The decrease in HLA-DR expression in intermediate monocytes during the chronic phase, although reminiscent of immune deactivation or monocyte immunoparalysis, may also reflect a return to baseline expression levels following acute inflammatory response.

Higher expression of CD163—a marker of macrophage activation—was observed in all circulating monocyte subsets. CD163 is a scavenger receptor involved in the clearance of free hemoglobin and is known for its anti-inflammatory properties, which are often associated with the resolution phase of inflammation [31]. In addition, patients in the chronic phase exhibit significantly elevated plasma levels of soluble CD163 (sCD163) in plasma. Furthermore, histopathological analysis of CHIKV-infected placentas by Salomão et al. revealed an increase in CD163+ cells, along with the presence of pro-inflammatory cytokines (e.g., IFN-γ and TNF-α) and anti-inflammatory cytokines (e.g., TGF-β and IL-10), suggesting that CD163+ macrophages may play a modulatory role in the immune response during CHIKV infection and potentially contribute to adverse pregnancy outcomes [61]. In dengue, a disease with clinical features overlapping with chronic CF, the sCD163 levels have been shown to be significantly elevated in severe cases compared to mild cases, thereby supporting its use as a potential biomarker for disease severity [62]. Additionally, increased sCD163 levels during the defervescence phase of dengue have been linked to disease progression, and reinforces its prognostic value [63]. Although our cohort did not include severe CHIKV cases, we longitudinally followed the patients from the acute/post-acute to the chronic phase (T1 vs. T2). Sustained elevation of sCD163 levels was observed over time, suggesting prolonged monocyte/macrophage activation and unresolved inflammation. Consistent with these plasma findings, the decrease in CD163 expression in non-classical monocytes between T1 and T2 likely reflects proteolytic cleavage and ongoing activation.

Elevated gene expression of several TLRs—including TLR1, TLR2, TLR3, TLR5, and TLR7/8—has been reported in patients naturally infected with CHIKV [33]. We reported that monocytes from dengue patients, as well as DENV-2-infected monocytes in vitro, exhibit increased expression of TLR2 and TLR4, which supports the involvement of TLR activation during infection [54]. Our results from the present study indicated that, during the acute phase of infection, intermediate (CD14+CD16+) and non-classical (CD14+CD16++) monocytes exhibited increased TLR7 expression. Additionally, the nonclassical monocytes exhibited higher TLR4 expression, which suggested differential activation patterns among the monocyte subsets. Valdés-López et al. demonstrated that TLR4 activation enhances IL-27 gene expression in CHIKV-infected macrophages, inducing a strong STAT1-dependent pro-inflammatory response independent of type I interferons [64]. Similarly, Felipe et al. revealed that CHIKV activates TLR2, TLR7, and TLR8 in infected monocytes, consequently triggering a rapid pro-inflammatory cytokine response within the first 6 h, which is essential for early viral control [15]. PCA indicated that TLR7-expressing intermediate and non-classical monocytes were associated with the chronic phase of the disease. Consistent with this finding, patients presenting with arthritis exhibited significantly increased frequencies of TLR7+ intermediate monocytes, thereby suggesting a potential role of this subset in the persistence of inflammation. CD16+ monocytes, including intermediate and non-classical subsets, exert strong inflammatory activity and serve as major producers of inflammatory mediators during viral infections [18]. TLR7, in turn, recognizes single-stranded viral RNA from arboviruses such as DENV and CHIKV, and triggers the production of type I interferons and pro-inflammatory cytokines [65]. However, persistent TLR7 activation has been implicated in the pathogenesis of chronic inflammatory and autoimmune diseases, wherein it may contribute to sustained immune activation and tissue damage [66]. Thus, TLR7 appears to play a dual role, whereby it is essential for antiviral defense, but is potentially pathogenic when chronically stimulated. Additionally, host genetic factors may influence TLR-mediated responses. Polymorphisms in TLR7 and TLR8 have been associated with increased susceptibility to CHIKV infection and modulation of cytokine production [67]. Although our data suggested a potential link between TLR7 expression and persistent inflammation, further studies are required to elucidate the mechanistic basis of this association.

Consistent with previous studies [35,68], our data revealed a pro-inflammatory profile characterized by significantly elevated levels of IL-6, TNF-α, and IL-17A during the acute phase. IL-17A—which is primarily produced by Th17 cells—has been implicated in the persistence of joint inflammation and may contribute to the transition from acute to chronic rheumatologic symptoms [36]. Moreover, the cytokines IL-6, IL-10, TNF-α, and IL-17A were positively correlated, suggesting a mixed functional profile and indicating that there were simultaneously inflammatory activation and regulatory/immunosuppressive components. Although some studies have reported sustained IL-10 elevation in chronic cases, especially in individuals with persistent arthralgia [19,68] a paired-sample analysis has revealed a general decline in IL-10 concentrations during the chronic phase. This heterogeneous pattern is consistent with previous reports of patients with persistent symptoms [19,68,69]. In particular, we found a direct correlation between the IL-10 levels and the frequency of intermediate monocytes. Combined with the existing evidence that this subset can produce IL-10 [70], these findings suggest a possible contribution of these cells to the anti-inflammatory milieu. Notably, we have previously observed increased indoleamine 2,3-dioxygenase 1 (IDO-1) activity during the acute phase of CHIKV infection [7]. IDO-1 is a key immunoregulatory enzyme involved in the induction and function of regulatory T cells (Tregs). Its expression in monocytes can be induced by inflammatory cytokines such as IFN-γ, as well as by IL-10 and TGF-β under certain conditions [71,72].

Although it is normally restricted to intracellular compartments, TF can be induced by pro-inflammatory cytokines such as TNF-α and IL-6, both of which are elevated during CHIKV infection and activated via TLR signaling [73]. Monocytes are recognized as the main source of circulating TF [38] and their upregulation contributes to thrombus formation, which serves as a host defense mechanism aimed at containing pathogens [74]. TF expression on monocytes/macrophages has been implicated in the coagulopathy observed in Ebola virus (EBOV) infection, where inhibition of the TF–FVIIa complex reduced mortality in infected non-human primates [75]. Similarly, monocytes from patients with severe DENV infection express higher levels of TF [76], and the circulating levels of TF and TFPI are correlated to the clinical severity of the infections caused by DENV-2 [41]. In the context of COVID-19, elevated TF expression in monocytes has been reported in severe cases, suggesting that viral infections can broadly activate coagulation pathways via innate immune mechanisms [77].

Our findings demonstrated increased levels of coagulation-related markers, including TF, in the context of CHIKV infection, which may indicate the activation of coagulation pathways. Although hemorrhagic events and thrombocytopenia were rare, previous studies have reported mild hemorrhagic manifestations such as petechiae and epistaxis in CHIKV-infected individuals [78,79], and platelet activation has been linked to disease chronicity [80]. Moreover, postural hypotension, a symptom reported during CHIKV infection, has been associated with vascular dysfunction in murine models infected by CHIKV [81].These findings suggest that inflammation-induced changes in coagulation contribute to the clinical manifestations of CHIKV infection. Consequently, we performed multivariate regression analyses to assess whether the coagulation markers independently predicted chronic joint symptoms. However, no statistically significant associations were found, likely due to the limited sample size. Nevertheless, this study provides further evidence of increased TF and TFPI concentrations in the circulation of CHIKV-infected patients and suggests a potentially novel mechanism for chikungunya pathology that warrants investigation in larger cohorts.

The persistent activation of pattern recognition receptors, particularly TLR7, is associated with both chronic inflammation and endothelial dysfunction [82]. TLR7 expression is generally higher in females and has been associated with stronger antiviral immunity, as well as increased susceptibility to chronic inflammatory and autoimmune conditions [83]. Although no significant sex-based differences were observed for most of the immune markers, female patients exhibited lower circulating TFPI levels. TFPI concentrations may vary with age and hormonal status; however, platelet full-length TFPI-alpha in healthy volunteers was reportedly not affected by sex or hormonal use [84]. Estradiol levels were higher in pre-menopausal women who exhibited lower TFPI concentrations, whereas post-menopausal women demonstrated the opposite pattern. These observations support the hypothesis that fluctuations in sex hormones, particularly estradiol, may influence coagulation dynamics and susceptibility to thromboinflammatory responses in CHIKV infections. Nonetheless, further studies are warranted to confirm these associations and explore their clinical relevance.

GFs such as VEGF, PDGF-BB, and EGF have been implicated in the pathogenesis of CF. Increased plasma levels of these mediators were found during the acute and chronic phases of chikungunya, thereby corroborating previous findings [68,85]. Interestingly, GFs are also known to play key roles in the pathogenesis of RA [86]. The VEGF concentrations, in particular, were significantly higher in patients with edema. Furthermore, the EGF levels were increased in patients with arthritis and positively correlated with TNF-α expression. Evidence from animal models of RA supports the functional role of GFs in joint inflammation and remodeling. For instance, VEGF promotes synovial neovascularization in collagen-induced arthritis, and its blockade attenuates disease severity in mice [87]. Additionally, the inhibition of the EGF receptor has been shown to reduce synovial inflammation and joint degradation in murine models of RA [88]. These findings align with our current observations, wherein elevated levels of VEGF and EGF were associated with edema and arthritis, thereby suggesting shared mechanisms of immune-mediated joint pathology.

Notably, other cell types—such as activated platelets [80], endothelial cells, and activated NK cells [13]—may also contribute to the circulating levels of inflammatory mediators and GFs. A limitation of our study was the absence of intracellular staining for cytokines and TF in monocytes, which precludes the definitive identification of their cellular sources. Nevertheless, phenotypic characterization of TLR4 and TLR7 expression in monocyte subsets supports their contribution to cytokine production and systemic inflammation during CHIKV infection. Another limitation was the lack of longitudinal follow-up to confirm the prognostic value of sCD163 and TLR7+ monocytes. Subgroup analyses revealed no significant differences in immunological parameters between young and elderly patients, or between those with and without comorbidities, indicating that age and comorbidities did not markedly affect immune activation in this cohort. Overall, our findings highlight the immune parameters that warrant evaluation in larger prospectively followed-up cohorts to assess their potential as predictive biomarkers of CHIKV infection. Future studies with extended follow-ups are essential to validate these findings and explore their utility as prognostic indicators or therapeutic targets in both the acute and chronic phases of the disease.

## 5. Conclusions

In summary, our data revealed marked activation of CD16+ monocytes and elevated levels of inflammatory mediators during acute CHIKV infection. Although the chronic phase findings require confirmation in larger cohorts, these results underscore the central role of monocyte-driven inflammation in chikungunya pathophysiology. Future studies should focus on delineating the cellular sources of each marker, as well as on incorporating longitudinal follow-ups for their cohorts. Ultimately, further research into these mechanisms underlying chikungunya pathology will reveal more efficient therapeutic markers and strategies for managing the disease.

## Figures and Tables

**Table 2:** Frequency of clinical manifestations in the acute/post-acute phase of patients with CHIKV infection according to sex.

Articular Manifestations	Total n = 93	Male 39	Female 54	OR (95% CI)	*p* Value
Arthralgia *		35 (89.7%)	52 (96.3%)	2.91 (0.65–16.0)	0.2334
Myalgia		31 (79.4%)	42 (77.7%)	0.90 (0.31–2.50)	>0.999
Arthritis		12 (30.7%)	17 (31.4%)	1.03 (0.41–2.45)	>0.999
Prostration		27 (62.9%)	41 (75.9%)	1.42 (0.56–3.44)	0.4875
Edema		14 (35.8%)	37 (68.5%)	3.88 (1.66–8.94)	0.0029
Low back pain		20 (51.2%)	37 (68.5%)	2.06 (0.89–4.97)	0.1308
**Extra-articular manifestations**					
Loss of appetite		20 (51.2%)	33 (61.1%)	1.49 (0.66–3.41)	0.3991
Nausea		15 (38.4%)	29 (53.7%)	1.85 (0.83–4.39)	0.2066
Exanthema		20 (51.2%)	30 (55.5%)	1.88 (0.53–2.66)	0.8333
Pruritus		17 (43.5%)	24 (44.4%)	1.03 (0.46–2.35)	>0.999
Headache		24 (61.5%)	42 (77.7%)	2.18 (0.89–5.40)	0.1079
Vertigo		8 (20.5%)	19 (35.1%)	2.10 (0.78–5.51)	0.1650
Retro-orbital pain		15 (38.4%)	27 (50.0%)	1.60 (0.71–3.77)	0.2977

OR: Odds Ratio; 95%CI: Confidence Interval; Fisher Exact Test, * *p* > 0.05.

## Data Availability

The original contributions presented in this study are included in the article/Supplementary Material. Further inquiries can be directed to the corresponding author.

## Supplementary Materials

The following supporting information can be downloaded at https://www.mdpi.com/article/10.3390/v17091224/s1. Figure S1: Gating strategy used to identify monocyte subsets in human peripheral blood; Table S1: Spearman correlation between cellular parameters and immunological markers in Chikungunya Fever (CF) patients during acute/post-acute phase; Table S2: Subtypes of Monocytes and Inflammatory Biomarker Levels in Chikungunya Fever (CF) Patients according sex; Table S3: Subtypes of Monocytes and Inflammatory Biomarker Levels in Chikungunya Fever (CF) Patients With or Without Articular Edema Compared to Healthy Controls. Table S4: Subtypes of Monocytes and Inflammatory Biomarker Levels in Chikungunya Fever (CF) Patients With or Without Arthritis Compared to Healthy Controls.
