# Supplementary material for: Monocyte Dynamics in Chikungunya Fever: Sustained Activation and Vascular-Coagulation Pathway Involvement
Source: Viruses. 2025 Sep 7;17(9):1224. doi: 10.3390/v17091224 (PMC12474508; doi:10.3390/v17091224)
Supplement: Supplementary file 1 [file viruses-17-01224-s001.zip › viruses-3699046-supplementary.pdf]

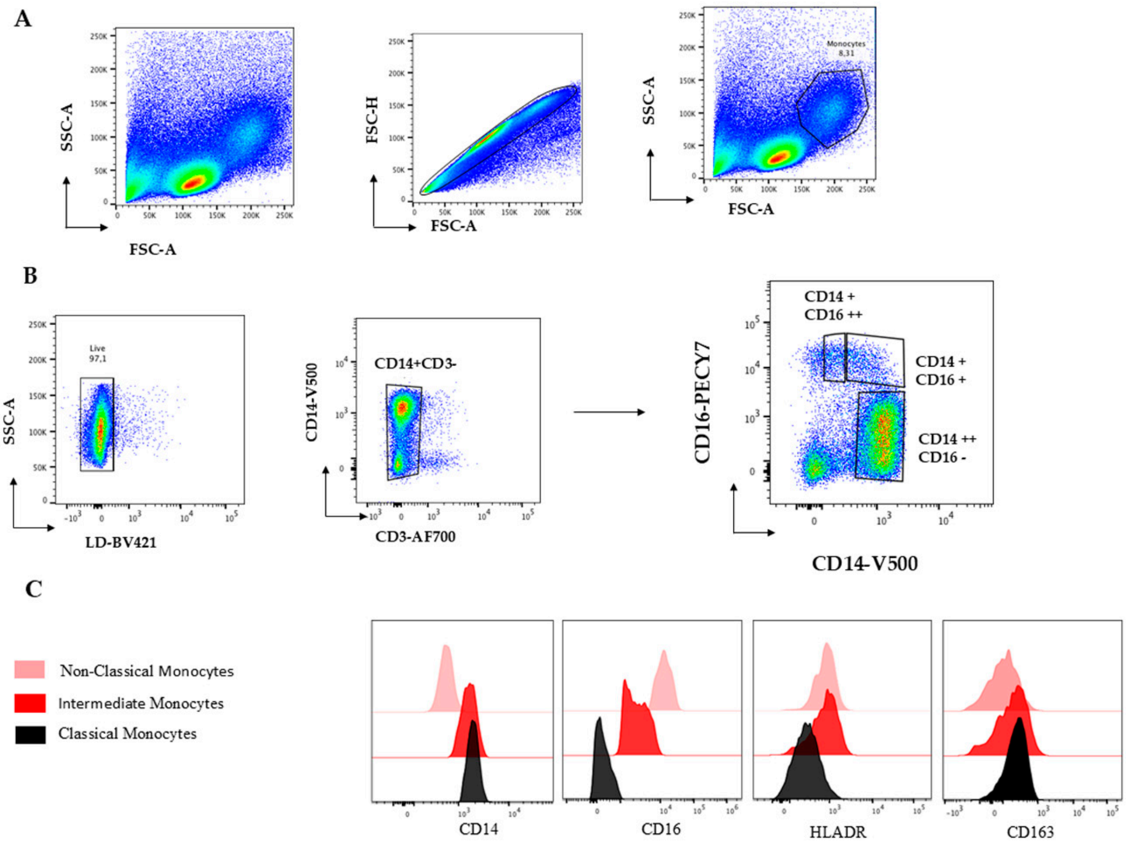

Supplementary Figure S1. Gating strategy used to identify monocyte subsets in human peripheral blood. (A) Flow cytometry density plots showing side scatter (SSC) vs. forward scatter (FSC) for morphological gating, doublet exclusion, and monocyte identification. (B) A viability gate using LB-BV421 was applied, followed by exclusion of CD3+(Alexa700) lymphocytes. Monocytes were identified based on CD14 and CD16 expression and classified into classical (CD14++CD16-), intermediate (CD14+CD16+), and non-classical (CD14+CD16++) subsets. (C) Histogram overlays displaying expression profiles of CD14-V500, CD16-PECy7, HLA-DR-PE-CY5, and CD163-BV-640 across the three monocyte subsets. Classical monocytes are shown in black, intermediate in red, and non-classical in pink.

Supplementary Table S1. Spearman correlation between cellular parameters and immunological markers in Chikungunya Fever (CF) patients during acute/post-acute phase.

Variable 1	Variable 2	p-valor	R
**CD14+CD16-HLADR**	CD14+CD16++HLADR	0.004	0.6
**CD14+CD16-HLADR**	CD14+CD16+HLADR	0.004	0.6
**CD14+CD16-HLADR**	CD14+CD16+HLADR	0,0001	0.7
**CD14+CD16-HLADR**	CD14+CD16+CD163	0.009	0.5
**CD14+CD16-CD163**	CD14+CD16++CD163	0.0001	0.7
**CD14+CD16+HLADR**	CD14+CD16++HLADR	0.005	0.6
**CD14+CD16+HLADR**	CD14+CD16+CD163	0.0210	0.5
**CD14+CD16+CD163**	CD14+CD16++CD163	0,0001	0.7
**CD14+CD16+CD163**	CD14+CD16+HLADR	0.0208	0.5
**CD14+CD16++TLR7**	CD14+CD16-HLADR	0.0121	-0.5
**IL-10**	CD14+CD16+	0.0260	0.3
**IL-10**	CD14+CD16+CD163	0.0266	0.5
**TNF- α**	EGF	0.0329	0.4
**TNF- α**	IL-6	0.0329	0.3
**IL-6**	sCD163	0.0119	0.4

Only included correlations that were statistically significant ($p < 0.05$).

Supplementary Table S2. Subtypes of Monocytes and Inflammatory Biomarker Levels in Chikungunya Fever (CF) Patients according sex.

Category	Parameter	Male	Female	Total	P value*
**Monocyte Subset (%)**					
	**N**	9	14	23	
	CD14+CD16-	62 (53-67)	55 (46-62)		0.1639
	CD14+CD16+	10 (10-18)	11 (10-19)		0.4481
	CD14+CD16++	5.2(2.3-7.6)	2.0 (2.0-7.0)		0.4807
**Activation Makers (MFI)**					
	CD14+CD16-HLA-DR	576 (402-648)	582 (422-642)		0.8960
	CD14+CD16+HLA-DR	871 (703-1020)	940 (702-1321)		0.6433
	CD14+CD16++HLA-DR	871 (637-1180)	1085 (811-1350)		0.3361
	CD14+CD16-CD163	320 (264-504)	367 (288-455)		0.8775
	CD14+CD16+CD163	481 (353-642)	482 (380-678		0.8644
	CD14+CD16++CD163	402 (269-696)	349 (260-494)		0.4670
**Toll Like Receptor (MFI)**					
	CD14+CD16-TLR7	309(283-387)	314 (271-354)		0.6994
	CD14+CD16+TLR7	294 (279-511	435 (345-493)		0.5465
	CD14+CD16++TLR7	352 (253-570)	533 (394-855)		0.2065
	N	7	8	15	
	CD14+CD16-TLR4	355 (275-387)	326 (379-375)		0.8410
	CD14+CD16+TLR4	393 (323-422)	382 (317-397)		0.6339
	CD14+CD16++TLR4	331 (282-500)	335 (299-361)		>0.9999
**Cytokines (pg/mL)**					
	N	19	29	48	
	IL-6	13.6 (4.6-47.6)	10.8 (5.2-34.5)		>0.9999
	TNF- α	6.4 (2.5—17.9)	4.3 (2.1—10.3)		0.3710
	IL-10	13.3 (7.9—23.1)	9.9 (5.4—16.8)		0.3462
	IL-17A	1.9 (1.2—3.7)	1.4 (0.9—2.8)		0.2061
**Coagulation Factors (pg/mL)**					
	N	16	23	39	
	TF	7.2 (4.1—18.4)	11.1 (6.6—23.1)		0.1571
	TFPI	**325.8 (205.4-528.7)**	**231 (146.8-319)**		**0.0347**
**Growth Factors (pg/mL)**					
	N	21	34	55	
	VEGF	173 (105.7-258.6)	157 (64.9-335.9)		0.8199
	N	14	27	41	
	EGF	104 (42.4-141.4)	74.6 (34.1-192.4)		0.7598
	N	13	32	45	

	PDGF-BB	1423 (477.9-3841)	757 (565.7-1236)		0.2397
**sCD163 (pg/mL)**					
	N	23	32	55	
	sCD163	273564 (202755-349707)	412077 (209597-662119)		0.0669

N - number of patients.

Data are expressed as median and interquartile range. * $p < 0.05$, Mann Whitney test.

MFI - Mean Fluorescence Intensity.

Supplementary Table S3. Subtypes of Monocytes and Inflammatory Biomarker Levels in Chikungunya Fever (CF) Patients With or Without Articular Edema Compared to Healthy Controls.

Category	Parameter	A.With Edema	B.Without Edema	C.Heathy Donor	P value		
**Monocyte Subset (%)**					A x C	B x C	A x B
	**N**	15	8	10			
	CD14+CD16-	57 (49-64)	62 (49-65)	73 (70-78)	<0.0001	0.0074	>0.9999
	CD14+CD16+	17 (10-20)	11 (10-17)	3.5 (2.4-4.5)	<0.0001	0.0077	>0.9999
	CD14+CD16++	4.1 (2.1-7.8)	3.8 (2.2-4.5)	2.4 (1.3-3.8)	0.1423	0.4739	>0.9999
**Activation Makers (MFI)**							
	CD14+CD16-HLA-DR	576 (479-621)	528 (363-756)	469 (368-610)	0.6529	>0.9999	>0.9999
	CD14+CD16+HLA-DR	994 (747-1111)	718(608-1199)	931 (588-1276)	>0.9999	>0.9999	>0.9999
	CD14+CD16++HLA-DR	994 (814-1344)	928 (595-1313)	804 (556-864)	0.0173	0.3493	>0.9999
	CD14+CD16-CD163	355 (276-422	418 (286-615)	252 (194-286)	0.0308	0.0122	>0.9999
	CD14+CD16+CD163	507 (391-612)	453 (353-821)	300 (254-323)	0.0082	0.0104	>0.9999
	CD14+CD16++CD163	507 (391-612)	438 (263-590)	201 (145-482)	0.0003	0.0154	>0.9999
**Toll Like Receptor (MFI)**							
	**N**	15	8	10			
	CD14+CD16-TLR7	314 (275-356)	322 (296-366)	241 (181-277)	0.0225	0.0136	0.0008
	CD14+CD16+TLR7	456 (359-525)	294 (272-465)	281 (182-358)	0.0115	>0.9999	0.1893
	CD14+CD16++TLR7	539 (352-850)	432 (223-576)	201 (145-346)	0.0003	0.0154	>0.9999
	**N**	10	5	10			
	CD14+CD16-TLR4	296 (277-379)	356 (353-389)	275 (265-289)	0.2840	0.0087	0.3204
	CD14+CD16+TLR4	378 (301-403)	391 (366-403)	307 (287-327)	0.1787	0.0177	0.6722
	CD14+CD16++TLR4	316 (256-341)	367 (332-488)	235 (210-267)	0.1912	0.0023	0.1934
**Cytokines (pg/mL)**							
	**N**	22	26	10			
	IL-6	10 (4.5-35.8)	10.3 (4.9-223)	1.9 (0.5-1.5)	<0.0001	0.0002	>0,9999
	TNF- α	6.3 (3.3—13)	4.4 (1.3—9.0)	1.2 (0.4-2.3)	0.0017	0.0275	0.70281

	IL-10	12 (7.0—21)	9.6 (3.9—16)	1.6 (1.1-4.2)	0.0002	0.0019	>0.9999
	IL-17A	2.0 (1.2—3.2)	1.5 (0.8—3.1)	1.0 (0.6-1.4)	0.0084	0.0763	0.8586
**Coagulation Factors (pg/mL)**							
	**N**	15	23	10			
	TF	8.4 (5.0—20)	11 (6.4—22)	3.8 (2.8-13)	0.2172	0.062	>0.9999
	TFPI	242 (163-334)	276 (178-407)	151 (86-203)	0.0998	0.0108	>0.9999
**Growth Factors (pg/mL)**							
	**N**	17	38	10			
	VEGF	291 (141-454)	141 (64-233)	67 (48-95)	0.0002	0.0558	**0.0239**
	**N**	11	30	10			
	EGF	192 (74-241)	87 (30-143)	79 (60-120)	0.3211	>0.9999	0.1777
	**N**	14	31	10			
	PDGF-BB	914 (645-2191)	757 (446-1405)	473 (127-729)	0.0218	0.0323	>0.9999
**SCD163 (pg/mL)**							
	**N**	27	28	10			
	sCD163	349707 (184644-512431)	312810 (210558-475980)	213894 (166968-263316)	0.1423	0.1177	>0.9999

N - number of patients.

Data are expressed as median and interquartile range. * $p < 0.05$, Kruskal–Wallis test followed by Dunn’s multiple comparisons test.

MFI - Mean Fluorescence Intensity.

Supplementary Table S4. Subtypes of Monocytes and Inflammatory Biomarker Levels in Chikungunya Fever (CF) Patients With or Without Arthritis Compared to Healthy Controls.

Category	Parameter	A.With Arthritis	B.Without Arthritis	C.Heathy Donor	P value		
**Monocyte Subset (%)**					A x C	B x C	A x B
	**N**	10	13	10			
	CD14+CD16-	58 (47-64)	59 (48-65)	73 (70-78)	0.0010	0.0005	>0.9999
	CD14+CD16+	17 (9.0-21)	11 (10-16)	3.5 (2.4-4.5)	<0.0001	0.0014	0.8711
	CD14+CD16++	6.9 (3.0-7.8)	2.5 (2.1-5.4)	2.4 (1.3-3.8)	0.0351	0.7859	0.3572
**Activation Makers (MFI)**							
	CD14+CD16-HLA-DR	570 (439-627)	595 (402-642)	469 (368-610)	0.9601	0.8686	>0.9999
	CD14+CD16+HLA-DR	1005 (702-1062)	866 (703-1345)	931 (588-1279)	>0.9999	>0.9999	>0.9999
	CD14+CD16++HLA-DR	889(769-1178)	1029 (710-1380)	804 (506-864)	0.1480	0.0301	>0.9999
	CD14+CD16-CD163	337 (288-426)	379 (264-571)	252 (194-286)	0.0400	0.0133	>0.9999
	CD14+CD16+CD163	449 (380-573)	507 (353-774)	300 (262-314)	0.0260	0.0017	>0.9999
	CD14+CD16++CD163	363 (258-473)	390 (266-571)	201 (145-223)	0.0190	0.0019	>0.9999
**Toll Like Receptor (MFI)**		10	13	10			
	**N**						
	CD14+CD16-TLR7	321 (275-375)	314 (275-361)	236 (174-263)	0.0030	0.0036	>0.9999
	CD14+CD16+TLR7	487 (415-532)	304 (286-475)	281 (182-358)	0.0048	0.6968	**0.0245**
	CD14+CD16++TLR7	538 (418-777)	414 (223-632)	201 (145-346)	0.0029	0.0547	0.7585
	**N**	6	9	10			
	CD14+CD16-TLR4	280 (269-432)	355 (326-373)	275 (265-289)	0.2449	0.0136	0.6380
	CD14+CD16+TLR4	357 (301-446)	390 (359-410)	307 (287-327)	0.2449	0.0317	>0.9999
	CD14+CD16++TLR4	298 (179-371)	341 (329-385)	235 (210-267)	0.8533	0.0018	0.1566
**Cytokines (pg/mL)**							
	**N**	18	30	10			
	IL-6	24 (6.2-55)	10 (5.8-25)	1.1 (0.5-1.5)	<0.0001	0.0001	0.9017
	TNF-α	9.3 (3.6—14)	3.7 (1.3—11)	1.2 (0.4-2.3)	0.0010	0.0250	0.1195
	IL-10	17 (7.0-35)	10 (4.4-15)	1.6 (1.1-4.2)	<0.0001	0.0027	0.3143
	IL-17A	2.4 (1.4—3.2)	1.5 (0.9—3.2)	1.0 (0.6-1.4)	0.0052	0.0503	0.6770
**Coagulation Factors (pg/mL)**							
	**N**	12	27	10			
	TF	11 (6.9—26)	10 (5.0—13)	3.8 (2.8-13)	0.0121	0.2602	0.2555
	TFPI	259 (171-342)	242 (155-407)	151 (86-203)	0.0708	0.0245	>0.9999
**Growth Factors (pg/mL)**							
	**N**	14	41	10			

	VEGF	176 (93-303)	149 (67-287)	67 (48-95)	0.0096	0.0086	>0.9999
	**N**	11	30	10			
	EGF	**163 (76-198)**	60 (33-129)	79 (60-120)	0.2381	>0.9999	**0.0223**
	**N**	10	35	10			
	PDGF-BB	1020 (645-1789)	757 (446-1423)	473 (127-729)	0.0249	0.0288	>0.9999
**Soluble Macrophage-Specific Scavenger Receptor (pg/mL)**							
	**N**	14	41	10			
	sCD163	339626 (195974-701419)	297466 (204703-543039)	213894 (166968-263316)	0.0892	0.1039	>0.9999

N - number of patients.

Data are expressed as median and interquartile range. * $p < 0.05$, Kruskal–Wallis test followed by Dunn’s multiple comparisons test.

MFI - Mean Fluorescence Intensity.
